# Autophagy: A Player in response to Oxidative Stress and DNA Damage

**DOI:** 10.1155/2019/5692958

**Published:** 2019-07-29

**Authors:** Serena Galati, Christian Boni, Maria Carla Gerra, Mirca Lazzaretti, Annamaria Buschini

**Affiliations:** ^1^Centre for Molecular and Translational Oncology-COMT, University of Parma, Parma 43124, Italy; ^2^Department of Chemistry, Life Sciences and Environmental Sustainability, University of Parma, Parma 43124, Italy; ^3^Department of Medicine, General Pathology Section, University of Verona, Verona 37134, Italy; ^4^Department of Health Science and Technology, University of Aalborg, Aalborg 9220, Denmark

## Abstract

Autophagy is a catabolic pathway activated in response to different cellular stressors, such as damaged organelles, accumulation of misfolded or unfolded proteins, ER stress, accumulation of reactive oxygen species, and DNA damage. Some DNA damage sensors like FOXO3a, ATM, ATR, and p53 are known to be important autophagy regulators, and autophagy seems therefore to have a role in DNA damage response (DDR). Recent studies have partly clarified the pathways that induce autophagy during DDR, but its precise role is still not well known. Previous studies have shown that autophagy alterations induce an increase in DNA damage and in the occurrence of tumor and neurodegenerative diseases, highlighting its fundamental role in the maintenance of genomic stability. During DDR, autophagy could act as a source of energy to maintain cell cycle arrest and to sustain DNA repair activities. In addition, autophagy seems to play a role in the degradation of components involved in the repair machinery. In this paper, molecules which are able to induce oxidative stress and/or DNA damage have been selected and their toxic and genotoxic effects on the U937 cell line have been assessed in the presence of the single compounds and in concurrence with an inhibitor (chloroquine) or an inducer (rapamycin) of autophagy. Our data seem to corroborate the fundamental role of this pathway in response to direct and indirect DNA-damaging agents. The inhibition of autophagy through chloroquine had no effect on the genotoxicity induced by the tested compounds, but it led to a high increase of cytotoxicity. The induction of autophagy, through cotreatment with rapamycin, reduced the genotoxic activity of the compounds. The present study confirms the cytoprotective role of autophagy during DDR; its inhibition can sensitize cancer cells to DNA-damaging agents. The modulation of this pathway could therefore be an innovative approach able to reduce the toxicity of many compounds and to enhance the activity of others, including anticancer drugs.

## 1. Introduction

Autophagy is a highly conserved catabolic pathway in eukaryotic cells, but its role is still controversial. What is certain is that it is necessary for cell survival and for the maintenance of homeostasis. In healthy cells, the pathway is activated at low basal levels, as a quality control pathway that eliminates long-lived or damaged proteins and organelles; it is also induced following different stressors to digest both intracellular and extracellular materials [1]. At the same time, under stress conditions, it can induce a programmed cell death, called “autophagy-dependent cell death” (ADCD) [2]. The autophagic pathway appears to be related to many biologic processes as aging, neurodegeneration, cardiovascular diseases, and cancer [3, 4].

Evidence shows autophagy activation also during the DNA damage response (DDR), through mTORC1 signaling [5–7]. Usually, damage to DNA induces several cellular processes; DDR enables cells either to eliminate or evade damage or to activate cell death pathways. Response to the DNA damage is mainly dependent on phosphorylation/dephosphorylation cascades driven by specific kinases as ATM (ataxia telangiectasia-mutated kinase), ATR (ataxia telangiectasia-mutated and Rad3-related protein), and the complex Rad17-RFC/9-1-1 complex (Rad9, Rad1, and Hus1). The 9-1-1 complex through Rad17 detects single-strand breaks on DNA (ss-DNA) and induces the activation of specific checkpoint signaling pathways. ATM and ATR are two serine/threonine kinases that control several processes as DNA replication, transcription, metabolic signaling, and DNA splicing. These kinases are able to counteract many proteins involved in cell cycle control (checkpoint kinases CHK1 and CHK2), cell survival (p53), genome surveillance (BRCA1), chromatin remodeling (HDAC1 and HDAC2), and regulation of DNA repair (FOXO3) [8]. It has been demonstrated that ATM has also a role in autophagy induction. As described by Stagni and collaborators, ATM activates the LKB1/AMPK/TSC2 signaling axis that culminates with the inhibition of the negative regulator mTOR complex 1 (mTORC1), resulting in autophagy induction through the activation of ULK1 (Unc-51-like autophagy activating kinase), which drives the nucleation and formation of the autophagosome membrane [9].

p53, a protein with a key role in genome stability and apoptosis induction, also seems to act as a regulator of the autophagic pathway. It can lead to autophagy during adverse growth conditions, keeping cells on a quiescent state. p53 also controls the switch from autophagy to apoptosis, through the regulation of the expression of autophagy (ULK and ATG family) and apoptosis- (Bcl2, PUMA, and Bax) related genes, depending on its activation signal. p53 phosphorylated on Ser15 induce p53/MDM2 dissociation, and free p53 inhibits Beclin1 and LC3, culminating in apoptosis activation and autophagy inhibition. In addition, p53 phosphorylated on Ser392 inhibits ULK1 directly, switching autophagy to apoptosis [10].

Alterations in autophagy have been shown to induce an increase in DNA damage and promote tumor and neurodegenerative disease occurrence, highlighting the importance of this pathway in maintenance of genomic stability [11]. Under DNA damage conditions, autophagy could act as a source of energy during cell cycle arrest and during repair mechanisms. On the other hand, autophagy seems to act also in degrading some components of repair machinery [12].

In the present study, in order to better understand the real role of autophagy in DNA damage response, we have evaluated the induction of autophagy in a histiocytic lymphoma cell line (U937) during the treatment with molecules which are able to induce DNA damage through different mechanisms of action (menadione, ethyl methanesulphonate (EMS), and bleomycin) or to induce a cell insult without affecting DNA integrity (bortezomib). U937 cells have the peculiarity to express many of the monocytic-like characteristics and were selected as a model cell line since autophagy plays an important role in acute leukemias [13], and in addition, this pathway seems to play a pivotal role in the growth and differentiation of this cell line [14]. Furthermore, the U937 cell line shows sensitivity to the drugs selected for this study [15–18].

Bleomycin is a radiomimetic antitumor antibiotic, widely used for the treatment of different cancers, namely, testicular cancer, lymphoma, lung cancer, cervical cancer, and cancers of the head and neck [19–21]. The best-known mechanism of action of this chemotherapeutic agent is the induction of DNA strand breaks, but bleomycin also seems to inhibit incorporation of thymidine into DNA strands. Bleomycin-mediated DNA degradation requires the presence of metal ions such as Fe^2+^ or Cu^+^ and molecular oxygen; the link between bleomycin and metal ions induces the formation of a pseudoenzyme that reacts with oxygen producing superoxide and hydroxide free radicals that cleave DNA. Bleomycin may also bind to specific sites in the DNA strand and induce breaks by extracting the hydrogen atom from the base, leading a Criegee-type rearrangement or the formation of an alkali-labile lesion, eventually resulting in DNA cleavage. This compound also mediates lipid peroxidation and oxidation of other cellular molecules [22]. BLM is able to induce ROS-mediated reticulum stress and autophagy in MCA205 (fibrosarcoma), B16F10 (melanoma) cell lines of C57BL/6 mice, and CT26 (colon carcinoma) cell line [23].

Ethyl methanesulphonate (EMS) is an alkylating agent with mutagenic, teratogenic, and carcinogenic properties [24]. It induces nucleotide substitution producing point mutations mainly. The principal base modification produced by EMS is the guanine alkylation to O^6^-ethylguanine leading to the transition mutation G:C to A:T [25, 26]. Alkylating agents promote RhoB phosphorylation and sumoylation, inhibiting mTORC1 activity, through the translocation of tuberous sclerosis complex (TSC complex) to lysosomes and then initiating autophagy [27].

Menadione, also named vitamin K3, is an organic compound whose principal mechanism of action is the generation of reactive oxygen species [28, 29]. Treatment with this compound induces cell growth inhibition and apoptosis in cancer cells. Apoptosis is induced via the reactive oxygen species-dependent mitochondria-related pathway [30–32]; the reactive oxygen species cause changes in mitochondrial membrane permeability, leading to the activation of caspases [33] and bringing the depletion of intracellular antioxidants such as glutathione (GSH). The depletion of GSH activates the apoptotic pathway [34]. Furthermore, menadione induces protein arylation [35]. Menadione induces autophagy and ER stress in Hela cells. Autophagy triggered by menadione prevents ER stress and the mitochondrial pathway of apoptosis [36].

**Figure 1:**
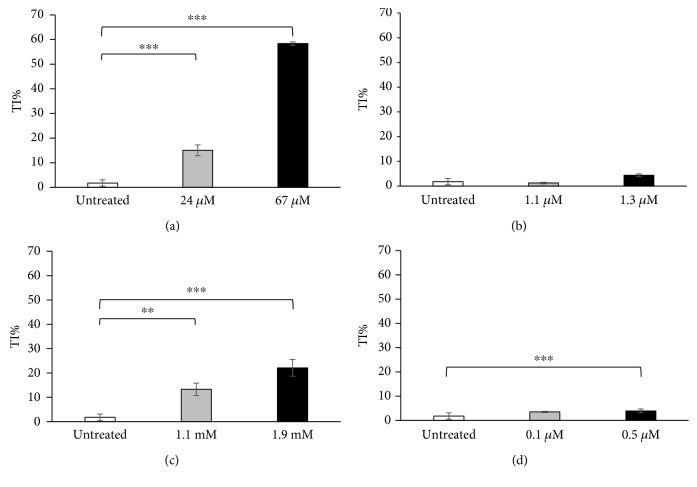
Genotoxicity evaluated through the comet assay in the U937 cell line after 24 h treatment with 24 and 67 *μ*M bleomycin (a), 0.1 and 0.5 *μ*M bortezomib (b), 1.1 and 1.9 mM ethyl methanesulphonate (c), and 1.1 and 1.3 *μ*M menadione (d). Data are given in terms of percentage of DNA in the comet tail (tail intensity percentage: TI%). The error bars represent the standard deviation of two independent experiments. ^∗^ *p* < 0.05, ^∗∗^ *p* < 0.01, and ^∗∗∗^ *p* < 0.001.

Bortezomib is the only molecule used in this work which is unable to induce DNA damage (Figure 1(b)). It is a proteasome inhibitor with antitumor activity against hematologic and nonhematologic malignancies [37]. Bortezomib could trigger autophagy enhancing the expression of autophagy-associated proteins LC3-II and Atg5–Atg12 complex and decreasing the expression of p62 in Hela and CaSki cells [38].

The toxic and genotoxic effects of the single compounds have been assessed on the U937 cell line, both alone and in combination with an inhibitor or an inducer of autophagy, chloroquine and rapamycin, respectively.

## 2. Materials and Methods

### 2.1. Chemicals

All chemicals were analytical grade, or they complied with the standards required for tissue culture experiments. Rapamycin, chloroquine, ethyl methanesulphonate, bortezomib, bleomycin, menadione, reagents for electrophoresis, normal melting point (1%) and low melting point (0.7%) agarose, dimethylsulfoxide, ethidium bromide, and general laboratory chemicals were from Sigma-Aldrich Company Limited (Milan, Italy). The cell culture medium and reagents were from BioWhittaker (Lonza, Milan, Italy).

### 2.2. Cell Lines

The U937 cells, a human histiocytic lymphoma, were obtained from American Type Culture Collection (Rockville, Maryland). They derived from malignant cells of a pleural effusion of a 37-year-old Caucasian male with diffuse histiocytic lymphoma. Nevertheless, they present the peculiarity to express many of the monocytic-like characteristics. The U937 cells were cultured in RPMI-1640 medium (Roswell Park Memorial Institute), supplemented with 10% (vol/vol) fetal bovine serum, 100 U/ml penicillin, 100 mg/ml streptomycin, and 2 mmol/l L-glutamine. Cells were maintained at 37°C in a humidified (95%) CO_2_ (5%) incubator and subcultured twice a week.

### 2.3. Cell Proliferation

Cell proliferation was detected by the CellTiter 96® AQ_ueous_ One Solution Cell Proliferation Assay (MTS) (Promega Corporation, Madison, WI, USA) as described by Ferrarini et al. [39]. This test contains a tetrazolium compound (MTS, inner salt) and an electron-coupling reagent (phenazine ethosulfate). The MTS tetrazolium compound is bioreduced by cells into a colored formazan product that is soluble in the culture medium. This conversion is accomplished by NADPH or NADH produced by dehydrogenase enzymes in metabolically active cells. In order to determine cell viability, in the exponential phase of growth, the cells were seeded at 5 × 10^4^/ml in ninety-six-well plates, in RPMI-1640 supplemented with 1% glutamine, 1% penicillin/streptomycin, and 5% fetal bovine serum. After seeding (24 h), U937 cells were treated, in quadruplicate, with increasing concentrations of the molecules and incubated for 24 h at 37°C in a humidified (95%) CO_2_ (5%) incubator. The cytotoxicity assay was performed by adding 20 *μ*l of the CellTiter 96® AQ_ueous_ One Solution Cell Proliferation Assay directly to culture wells, incubating for 4 h, and then recording the absorbance at 450 nm with a ninety-six-well plate reader (Multiskan EX; Thermo Electron Corporation, Vantaa, Finland).

The MTS assay was used to obtain a dose-effect curve for every compound, and according to Shoemaker [40], the concentrations able to inhibit the 10% (GI_10_) and the 50% (GI_50_) of the cell growth, the concentration that totally inhibits cell growth (TGI), and the 50% lethal concentration (LC_50_) were extracted from concentration-response curves by linear interpolation.

### 2.4. Evaluation of the Genotoxicity of Molecules on Human Cells

To assess primary DNA damage, the alkaline version of the comet assay was performed with U937 cells as described by Buschini et al. [41]. Briefly, the cells were seeded at a concentration of 2 × 10^5^ cell/ml in 24-well plates in 1 ml of RPMI-1640 (Roswell Park Memorial Institute), supplemented with 1% glutamine, 1% penicillin/streptomycin, and 10% fetal bovine serum and then incubated at 37°C in a humidified (95%) CO_2_ (5%) incubator. After 24 h, cells were treated, in duplicate, with the GI_10_ and GI_50_ of bleomycin, bortezomib, ethyl methanesulphonate, and menadione, calculated through the MTS assay. After 24 h of treatment, the determination of the cell number and viabilities was performed with the trypan blue exclusion method. DNA was stained with 75 *μ*l ethidium bromide (10 *μ*g/ml) before the examination at 400x magnification under a Leica DMLS fluorescence microscope (excitation filter BP 515-560 nm and barrier filter LP 580 nm), using an automatic image analysis system (Comet Assay IV, Perceptive Instruments Ltd., UK).

The total percentage of fluorescence in the tail (TI (tail intensity)) provided representative data on genotoxic effects. For each sample, coded and evaluated blind, 100 cells were analyzed and the median value of TI was calculated. At least two independent experiments were performed for each extract, and the mean of the median TI values was used for statistical analyses.

### 2.5. Autophagy Assessment

The formation of autophagic vesicles was assessed using a CYTO-ID Autophagy Detection Kit (Enzo Life Sciences, Farmingdale, NY), according to the manufacturer's instructions [42, 43]. This kit monitors autophagic flux in live cells using a novel dye that selectively labels autophagic vacuoles (preautophagosomes, autophagosomes, and autophagolysosomes). Rapamycin (0.1 *μ*M), a known autophagy inducer, was used as a positive control and chloroquine (10 *μ*M), an autophagy inhibitor, as a negative control. Autophagy analysis was performed by incubating cells with GI_10_ and GI_50_ of bleomycin, bortezomib, ethyl methanesulphonate, and menadione for 24 h at 37°C prior to treatment with the CYTO-ID Green Detection Reagent and analyzing fluorescence by flow cytometry using the NovoCyte Flow Cytometer (ACEA, Biosciences Inc.). For every condition, 20000 events were collected. In order to better evaluate the possible induction of autophagy with the different compounds, a cotreatment with the autophagy inhibitor (chloroquine), which is able to induce the accumulation of autophagosomes in the cytoplasm, was performed for every tested molecule.

**Figure 2:**
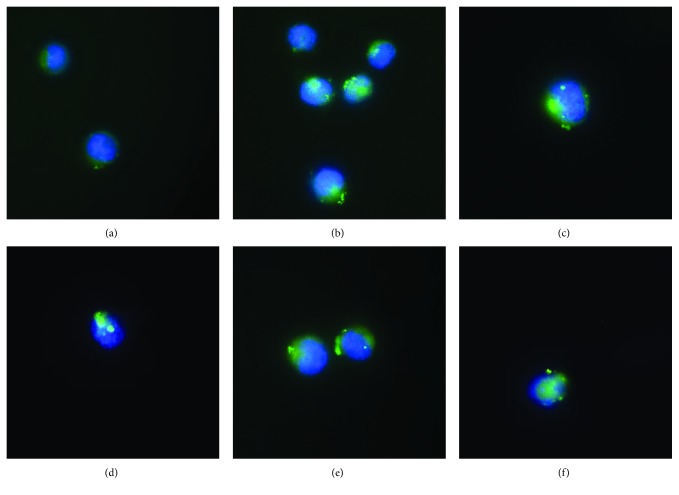
Autophagasome accumulation induction detected through a transfection protocol with the pEGFP-LC3 human plasmid in the untreated U937 cell line (a) after a 16 h treatment with 0.1 *μ*M rapamycin (b) and after a 24 h treatment with 1.9 mM ethyl methanesulphonate (c), 67 *μ*M bleomycin (d), 1.3 *μ*M menadione (e), and 0.5 *μ*M bortezomib (f).

Autophagic pathway activation has been also evaluated through a transfection protocol using a plasmid encoding the autophagosome marker LC3 fused with the fluorescent protein EGFP (pEGFP-LC3 human, Addgene). Transfection was performed using the Lipofectamine™ reagent (Invitrogen®), consisting of lipidic subunits that can form liposomes in an aqueous environment that entraps plasmid and drives it inside the cells. Transfection allows cells a constitutive LC3-EGFP fusion protein synthesis. Its nuclear and cytoplasmic distribution confers a uniform fluorescence to the cell; autophagy activation induces the formation of LC3-EGFP aggregates that determine the fluorescent signal amplification, conferring a punctuated morphology with the green spot in the cytoplasm exclusively (Figure 2).

For the assay execution, 2.5∗10^3^ cells were seeded in 24-well plates in 1 ml of growth medium and then incubated at 37°C in a humidified (95%) CO_2_ (5%) incubator. After 24 h, cells were transfected with the plasmid as described above according to the following protocol: 4 *μ*g of plasmid was diluted in 200 *μ*l of Opti-MEM (Invitrogen®) and at the same time, 5 *μ*l of Lipofectamine™ is gently mixed with 200 *μ*l Opti-MEM. After the first incubation for 5–10 min, the diluted plasmid solution and diluted Lipofectamine™ solution were gently mixed and incubated for 20 min to promote the formation of Lipofectamine™:plasmid complexes, 30 *μ*l of solution containing the Lipofectamine™:plasmid complexes was added to each well, and cells were incubated at 37°C in a humidified (95%) CO_2_ (5%) incubator for 24 h. At the end of transfection, cells were treated with the GI_50_ of the molecules for 24 h. After treatment, growth medium was removed; cells were washed twice with PBS, dropped on a glass slide, and then fixed in 400 *μ*l of fixative solution for 30 min at RT; fixative solution was removed; cells were washed three times with PBS; and cover slips were mounted onto slides using the VECTASHIELD mounting medium with DAPI. For the visualization of LC3-EGFP aggregates, cells were examined through a fluorescent microscope using an oil immersion objective (63x magnification). For each sample, 200 transfected cells were analyzed; in autophagy-negative cells, LC3-EGFP exhibits a diffuse cytoplasmic signal; when autophagy is induced, LC3-EGFP chimeric proteins aggregate in autophagic vacuoles, leading to a punctuate cytoplasmic staining [44].

### 2.6. Autophagic Pathway Modulation

In order to investigate the role of autophagy in the cell response to the stress induction, cells were cotreated with the GI_10_ and the GI_50_ of the tested molecules and an autophagic inhibitor (chloroquine, 3 *μ*M) or an activator (rapamycin, 0.1 *μ*M). Variations in terms of cytotoxicity and genotoxicity were evaluated through the MTS assay (see Cell Proliferation) and comet assay (see Evaluation of the Genotoxicity of Molecules on Human Cells), respectively.

### 2.7. Statistical Evaluation

The data were analyzed using the statistical and graphical functions of SPSS 25 (SPSS Inc., Chicago, IL, USA). Differences were assessed using ANOVA, followed by Bonferroni's post hoc test as appropriate, for parameters normally distributed such as means of optical density values and of median TI values. Significance was accepted at the *p* < 0.05 level.

## 3. Results

### 3.1. Cell Proliferation

Cell proliferation, detected through the MTS assay, allowed us to calculate GI_10_, GI_50_, TGI, and LC_50_ from a dose-effect curve (Table 1). A lethal concentration has been identified only for menadione; this molecule seems to induce a high toxicity at very low concentrations. A concentration that could induce a growth inhibition over 50% has not been found for EMS, even in assaying really high concentrations; as reported in literature, this compound is a potent mutagen with a low cytotoxic activity [45]. For bortezomib and bleomycin, a total growth inhibition concentration was identified but the 50% lethal concentration was not reached at the assayed concentrations.

### 3.2. DNA Damage Induction

The capability of the selected compounds to induce DNA damage after 24 h of treatment was evaluated through the alkaline version of the comet assay. The known genotoxic molecules (bleomycin, EMS, and menadione) showed an increase in tail intensity percentage in a dose-dependent manner (Figures 1(a), 1(c), and 1(d)). Bleomycin is the one that induced the higher DNA fragmentation, followed by EMS and lastly menadione. The relative low DNA damage observed after treatment with menadione is explained because the oxidative damage, the principal insult induced through treatment with this molecule, is one of the most rapidly repaired. Reactive oxygen species are a by-product of respiration, and cells remove them through antioxidant enzymes or scavengers such as glutathione and activating DNA repair mechanisms, like base excision repair. The damage measured is in a “dynamic steady state” [46]. As expected, no genotoxicity was observed after the treatment with bortezomib (Figure 1(b)).

### 3.3. Autophagic Pathway Induction

Induction of the autophagic pathway after treatment with the genotoxic drugs (bleomycin, EMS, and menadione) and the proteasome inhibitor (bortezomib) was revealed through a transfection assay; an increase in fluorescent dots was observed in U937 cells treated with all the assayed molecules and with the positive control rapamycin (Figure 2).

**Figure 3:**
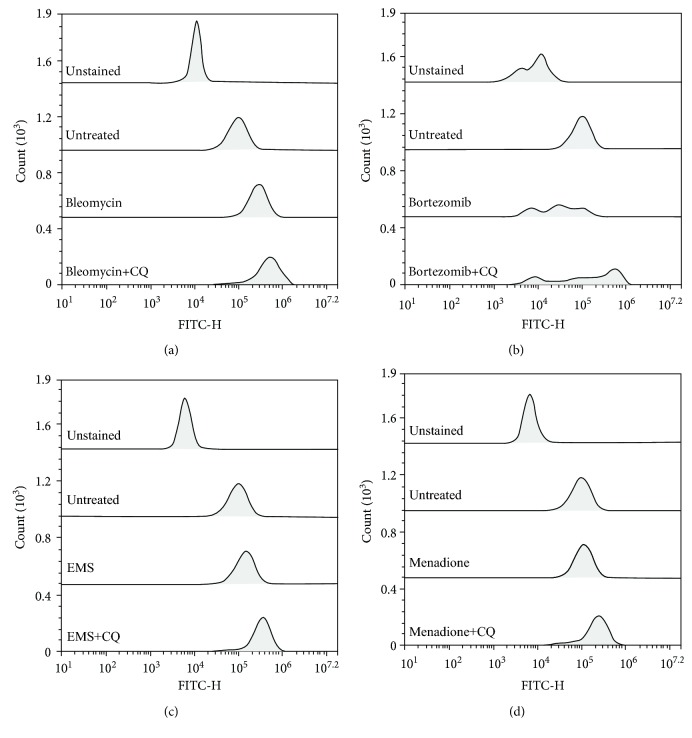
Autophagic pathway induction evaluated through flow cytometry in the U937 cell line after a 24 h treatment with 67 *μ*M bleomycin (a), 0.5 *μ*M bortezomib (b), 1.9 mM ethyl methanesulphonate (c), and 1.3 *μ*M menadione (d). The accumulation of autophagic vesicles was induced through the treatment with the autophagy inhibitor, 10 *μ*M chloroquine. 20000 events were collected for every tested condition through the NovoCyte Flow Cytometer (ACEA, Biosciences Inc.).

During the treatment with the tested molecules, the involvement of the autophagic pathway in cell response was also assessed through the CYTO-ID Autophagy Detection Kit. Also, in this case, all the tested compounds seemed to act as autophagic inducers in cells treated both with the GI_50_ (Figure 3) and with the GI_10_ (Figure S1, in Supplementary Materials).

The cotreatment of the single molecules with chloroquine induced an increase in the fluorescence signal, similar to that registered for cells cotreated with chloroquine and rapamycin (Figure S2, in Supplementary Materials), meaning the accumulation of autophagosomes in the cytoplasm (Figure 3).

Autophagy is a highly expressed pathway under stress conditions, such as following treatment with cell-damaging drugs. It is fundamental to understand the role of this pathway in response to every compound, because of its dual mode of action. Autophagy can act to promote cell survival, acting as a protein or organelle quality control mechanism, or during extremely stress condition, to induce intracellular toxicity and cell death [19].

### 3.4. Autophagic Pathway Modulation

In order to better understand the role of the autophagic pathway during the DNA damage response, cotreatments with the GI_50_ of the selected molecules and chloroquine, as an autophagy inhibitor, or rapamycin, as an autophagy activator, were performed. Variations in terms of cytotoxicity and genotoxicity, compared to those observed after the treatment with the single compounds, were evaluated through the MTS assay and comet assay.

**Figure 4:**
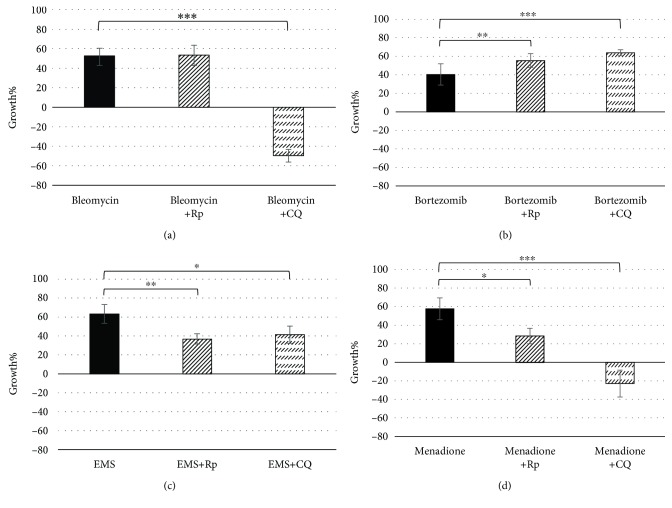
Cell proliferation evaluated through the MTS assay in the U937 cell line cotreated for 24 h with 0.1 *μ*M rapamycin or 3 *μ*M chloroquine and 67 *μ*M bleomycin (a), 0.5 *μ*M bortezomib (b), 1.9 mM ethyl methanesulphonate (c), and 1.3 *μ*M menadione (d). Data are given in terms of growth percentage (Growth%). The error bars represent the standard deviation of four independent evaluations. ^∗^ *p* < 0.05, ^∗∗^ *p* < 0.01, and ^∗∗∗^ *p* < 0.001.

**Figure 5:**
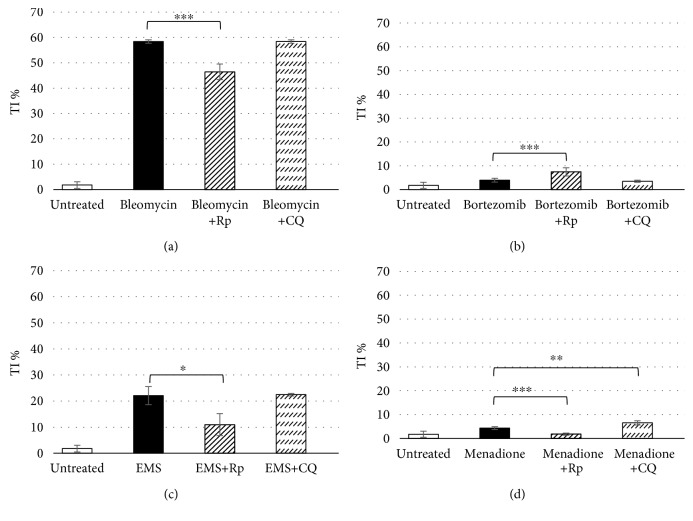
DNA damage evaluated through the comet assay in the U937 cell line cotreated for 24 h with 0.1 *μ*M rapamycin or 3 *μ*M chloroquine and 67 *μ*M bleomycin (a), 0.5 *μ*M bortezomib (b), 1.9 mM ethyl methanesulphonate (c), and 1.3 *μ*M menadione (d). Data are given in terms of percentage of DNA in the comet tail (tail intensity percentage: TI%). The error bars represent the standard deviation of two independent experiments. ^∗^ *p* < 0.05, ^∗∗^ *p* < 0.01, and ^∗∗∗^ *p* < 0.001.

The inhibition of autophagy during the treatment with the DNA-damaging molecules induced variations in terms of cell proliferation in the U937 cell line. Specifically, we have observed a high cytotoxic effect in the case of cells treated with bleomycin and menadione (Figures 4(a) and 4(d)) and a cytostatic one in those treated with ethyl methanesulphonate (Figure 4(c)). The inhibition induced an increase in DNA fragmentation detected through the comet assay only in U937 cells treated with menadione (Figure 5(d)), maybe due to the activation of apoptosis as a consequence of autophagy inhibition or to the major disposal of reactive oxygen species. The cotreatment with the autophagy inducer, rapamycin, determined a cytostatic effect in cells treated with ethyl methanesulphonate and menadione (Figures 4(c) and 4(d)), but there were no variation in terms of cell proliferation in U937 treated with bleomycin (Figure 4(a)). Interestingly, a reduction of DNA damage was observed after the activation of the autophagy by all three DNA-damaging compounds (Figures 5(a), 5(c), and 5(d)), confirming an involvement of the autophagic pathway in the maintenance of the DNA integrity.

The alteration of the autophagic pathway during the treatment with the proteasome inhibitor, bortezomib, induced an increase of cell proliferation (Figure 4(b)). This confirms the fundamental role of autophagy in the maintenance of cell cycle arrest induced through the treatment with bortezomib. The increase in DNA damage observed after autophagy induction (Figure 5(b)) could be due to the triggering of apoptosis that sometimes occurs following autophagy activation [47].

## 4. Discussion and Conclusion

In recent years, the scientific community has focused its attention on the autophagic pathway because of its involvement both in cellular homeostasis and in human pathologies, such as neurodegeneration, cardiovascular diseases, and cancer. At first, this pathway was studied as an alternative pathway of cell death; subsequently, it was revaluated and associated with mechanisms of repair and cell survival. Recently, its role in DNA damage response is extensively investigated.

In this work, we have selected four different molecules which are able to induce different kinds of cellular damages; in particular, three of these (bleomycin, EMS, and menadione) are known to induce different DNA damages, and the last one (bortezomib) is a known chemotherapeutic agent, acting as an inhibitor of the proteasome. For each compound, we have selected the concentrations which are able to reduce by 10% (GI_10_) and by 50% (GI_50_) of the cell growth (Table 1). These concentrations have been used to investigate cellular response in terms of genotoxicity and autophagic response. Through a preliminary comet assay, we have confirmed the induction of DNA damage after the treatment with bleomycin, EMS, and menadione, but not after the treatment with bortezomib, as expected (Figure 1). Activation of the autophagic pathway after treatments was revealed through different protocols: a transfection method using a plasmid encoding the autophagosome marker LC3 fused with the fluorescent protein EGFP (Figure 2) and a flow cytometric detection of the formation of autophagic vesicles through the CYTO-ID Autophagy Detection Kit (Figure 3).

Due to the great heterogeneity in the mechanisms of action of these four compounds, it has been indispensable to analyze each single molecule individually.

Bleomycin is a radiomimetic antibiotic with a complex mechanism of action including induction of DNA strand breaks and DNA oxidative damage. In our study, *in vitro* treatment of the U937 cell line with bleomycin induced, in addition to DNA damage (Figure 1(a)), a high level of autophagy, as shown in Figures 2(d) and 3(a); the cotreatment of the cells with bleomycin and chloroquine, the autophagy inhibitor, induced a high accumulation of autophagic vesicles (Figure 3(a)). The high level of autophagy might be explained in the light of the peculiar mechanism of action of bleomycin just described, such as the induction of oxidative stress and protein modification. The inhibition of autophagy through chloroquine during cell treatment with bleomycin induced a high reduction of cell viability (Figure 4(a)); this observation supports a prosurvival role for autophagy during the treatment with this molecule. The involvement of the autophagic pathway in the cell response to DNA damage is confirmed by the reduction of the fragmentation of DNA induced through bleomycin after the cotreatment with rapamycin, the autophagic inducer (Figure 5(a)). Autophagy seems to have a role in the protection of the cell after DNA clastogenic insult.

EMS is an alkylating agent inducing mainly gene mutation. Our data confirm the activation of the autophagic pathway in cells treated with this compound (Figures 2(c) and 3(c)). Autophagy modulation, through rapamycin or chloroquine, induced a reduction of cell proliferation (Figure 4(c)), confirming that autophagy is a finely controlled pathway. The capability of this molecule to induce alkylation, not only on DNA but also on proteins and cell structures, could be the principle responsible for autophagy induction. Autophagy could act as a cell scavenger, eliminating damaged and misfolded cellular components. Our data seem to confirm also its role in DNA damage response and repair; a reduction in DNA damage induced by EMS was observed after cotreatment with the autophagy inducer (Figure 5(c)), as already seen for bleomycin.

Menadione is a vitamin whose principal mechanism of action is the generation of ROS, resulting in DNA damage. This molecule was able to induce the autophagic pathway (Figures 2(e) and 3(d)). The inhibition of the autophagic pathway led to an increase of cytotoxicity (Figure 4(d)) together with an increase in DNA fragmentation (Figure 5(d)), maybe due to a major bioavailability of the reactive oxygen species, and finally to the activation of cell death pathways as apoptosis. The cell cotreatment with menadione-rapamycin leads to a decrease in cell proliferation (Figure 4(d)), followed by a reduction of the DNA damage (Figure 5(d)), as seen for bleomycin and EMS too.

Bortezomib is a proteasome inhibitor and is the only molecule used in this work without genotoxic activity (Figure 1(b)). Bortezomib seems to act as an autophagy inducer (Figures 2(f) and 3(b)), as expected, because of the cross talk existing between the two intracellular degradation systems, the ubiquitin-proteasome system (UPS) and the autophagy [48]. Inhibition of the UPS leads to autophagy activation [49–51], and autophagy inhibition enhances UPS activity [52]. The contextual cell treatment with bortezomib and chloroquine or rapamycin induced an increase in cell proliferation (Figure 4(b)), maybe due to the loss of cell cycle arrest in the G2/M phase induced by bortezomib [53–55]. This could confirm the involvement of the autophagic pathway in the antimitogenic action of proteasome inhibitors [51]. No variations in terms of DNA damage were observed after cotreatment with chloroquine, but autophagy activation induced DNA fragmentation (Figure 5(b)). The appearance of fragmented DNA could be due to the activation of apoptosis. Usually, autophagy inhibits this cell death pathway, and apoptosis-associated caspase activation switches off the autophagic process, but it has been reported that sometimes the induction of autophagy facilitates the activation of apoptosis [47]. Proteins that have an essential role in autophagy may have proapoptotic activity; many intracellular signal pathways induced by stress regulate both autophagy and apoptosis, explaining the sequential activation of both processes. In the presence of high stress conditions such as ionizing radiation, chemotherapeutic agents, and inhibition of growth factor receptors or starvation, autophagy is rapidly induced as a mechanism to adapt to stress, and subsequently, it is followed by the activation of cell death pathways [56, 57]. In particular, during the treatment with bortezomib, the autophagosome formation activates caspase 8, and consequently, the activation of the effector caspase 3 is detectable [58]. Moreover, autophagy may deplete endogenous inhibitors of apoptosis [47]. Finally, the hyperactivation of autophagy could induce the elimination of fundamental proteins for the maintenance of genomic stability [59].

The data reported in this study show a different behavior of the autophagic pathway in the U937 cell line treated with DNA-damaging molecules versus those treated with bortezomib. In the first case, the inhibition of autophagy induces cytotoxicity, especially in cells treated with bleomycin and menadione, confirming the protective role of autophagy in response to external stressors, as reported in the literatures [60–62]. On the contrary, in cells treated with bortezomib, the inhibition of autophagy induces an increase in cell proliferation. The most interesting observations come from cells cotreated with the autophagy inducer rapamycin; a reduction in DNA damage induced by the single treatment has been observed in all the treatments with the three DNA-damaging molecules, associated with a reduction of cell proliferation only after treatment with EMS and menadione. Literature data show that autophagy has an important role in the prevention of DNA damage; autophagy-deficient cancer cells accumulate *γ*-H2aX foci and genome damage, leading to tumor progression [63]. The real role of autophagy in DNA integrity maintenance is still unclear, but it could play a vital role in the elimination of proteins, organelles, and damaged DNA or in the recovery of ATP, essential for cellular functions required to maintain genome integrity, such as mitosis, DNA replication, and repair [64–66]. Autophagy is able to limit cellular damage through the maintenance of energy homeostasis, oxidative stress reduction, and elimination of damaged proteins and organelles [67]. Deficient autophagy compromises the cell adaptation to metabolic stress, including insufficient ATP generation and accumulation of damaged mitochondria [68].

In conclusion, we have observed a similar behavior during the autophagy modulation in cells treated with the DNA-damaging molecules; the induction of autophagic pathway enables cells to partially recover the DNA damage. In the presence of molecules which are able to induce oxidative stress (i.e., bleomycin and menadione), the inhibition of autophagy induces high cytotoxicity. These data confirm the important role of autophagy in cell response to genotoxic stress. Modulation of autophagy appears to be a successful approach to reduce toxicity or to enhance the activity of different molecules, including anticancer drugs.

## Figures and Tables

**Table 1:** GI_10_ (10% growth inhibition), GI_50_ (50% growth inhibition), TGI (total growth inhibition), and LC_50_ (50% lethal concentration) extracted from the concentration-response curves by linear interpolation.

	Compound			
	Bleomycin	Bortezomib	EMS	Menadione
GI_10_	24 *μ*M	0.1 *μ*M	1.2 mM	1.1 *μ*M
GI_50_	67 *μ*M	0.5 *μ*M	1.9 mM	1.3 *μ*M
TGI	100 *μ*M	2.4 *μ*M	>2 mM	1.7 *μ*M
LC_50_	>100 *μ*M	>10 *μ*M	>2 mM	1.9 *μ*M

## Data Availability

All the data used to support the findings of this study are included within the article.

## Supplementary Materials

Supplementary Materials Supplementary materials integrate data described in the main document. In particular, Figure S1 of supplementary materials shows the induction of autophagy, evaluated through flow cytometry, in the U937 cell line treated for 24 h with the GI_10_ of the assayed compounds (i.e., 24 *μ*M bleomycin, 0.1 *μ*M bortezomib, 1.1 mM ethyl methanesulphonate, and 1.1 *μ*M menadione). Figure S2 reports the flow cytometric analysis of cells treated with rapamycin (0.1 *μ*M), chloroquine (10 *μ*M), and rapamycin and chloroquine together to detect the accumulation of autophagic vesicles in the cytoplasm.
