# Supplementary material for: Autophagy: A Player in response to Oxidative Stress and DNA Damage
Source: Oxid Med Cell Longev. 2019 Jul 29;2019:5692958. doi: 10.1155/2019/5692958 (PMC6701339; doi:10.1155/2019/5692958)
Supplement: Supplementary Materials — integrate data described in the main document. In particular, Figure S1 of supplementary materials shows the induction of autophagy, evaluated through flow cytometry, in the U937 cell line treated for 24 h with the GI10 of the assayed compounds (i.e., 24 μM bleomycin, 0.1 μM bortezomib, 1.1 mM ethyl methanesulphonate, and 1.1 μM menadione). Figure S2 reports the flow cytometric analysis of cells treated with rapamycin (0.1 μM), chloroquine (10 μM), and rapamycin and chloroquine together to detect the accumulation of autophagic vesicles in the cytoplasm. [file 5692958.f1.pdf]

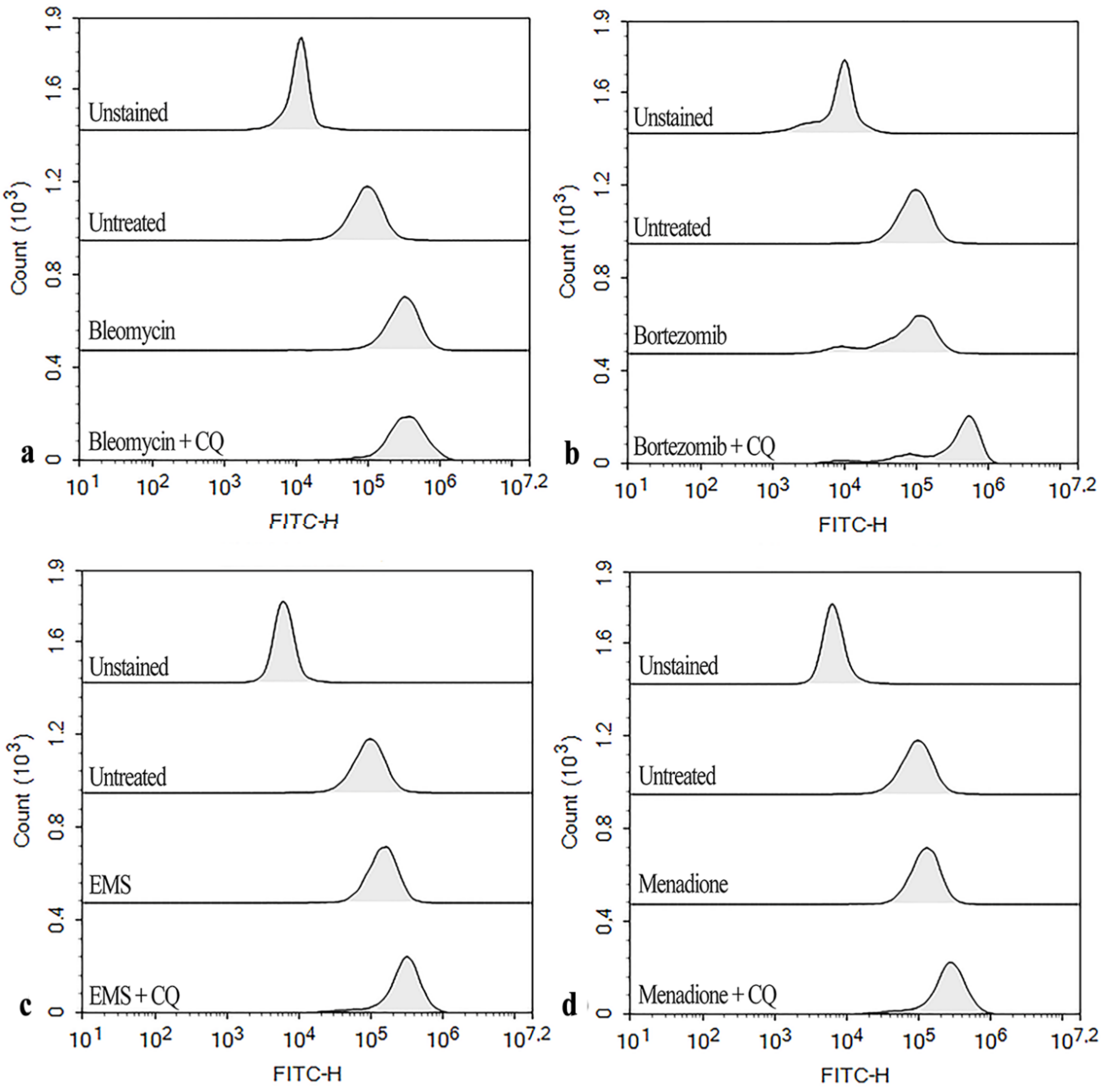

Figure 1S: Autophagic pathway induction evaluated through flow cytometry in U937 cell line after a 24h treatment with 24 µM Bleomycin (a), 0.1 µM Bortezomib (b), 1.1 mM ethyl methansulphonate (c) and 1.1 µM Menadione (d). The accumulation of autophagic vesicles were induced trough the treatment with the autophagy inhibitor, 10 µM chloroquine. 20000 events were collected for every tested condition through the NovoCyte Flow Cytometer (ACEA, Biosciences, Inc.).

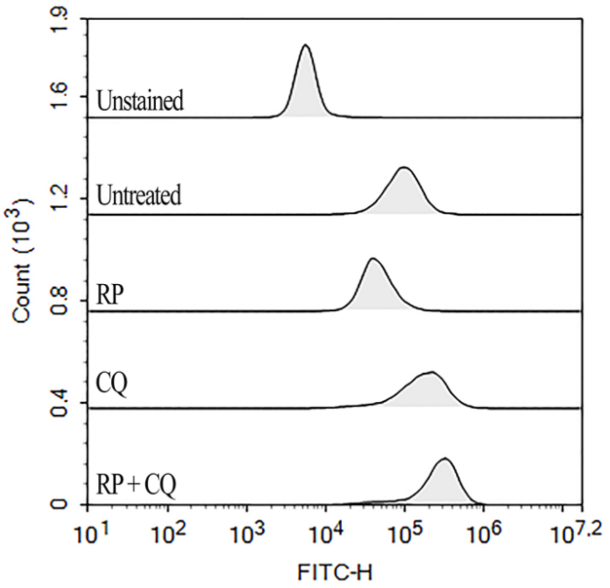

Figure 2S: Autophagic pathway induction evaluated through flow cytometry in U937 cell line after 18h treatment with 10 µM chloroquine and 0.1 µM rapamycin. The co-treatment with chloroquine and rapamycin is performed to confirm that chloroquine, inducing the autophagic vesicles accumulation in the cytoplasm, allows to detect the action of rapamycin in triggering autophagic vesicles development. 20000 events were collected for every tested condition through the NovoCyte Flow Cytometer (ACEA, Biosciences, Inc.).
